# The effect of impedance-controlled robotic gait training on walking ability and quality in individuals with chronic incomplete spinal cord injury: an explorative study

**DOI:** 10.1186/1743-0003-11-26

**Published:** 2014-03-04

**Authors:** Bertine M Fleerkotte, Bram Koopman, Jaap H Buurke, Edwin H F van Asseldonk, Herman van der Kooij, Johan S Rietman

**Affiliations:** 1 Roessingh Research and Development, Enschede, The Netherlands; 2 Institute for Biomedical Technology and Technical Medicine (MIRA), Department of Biomechanical Engineering, University of Twente, Enschede, The Netherlands; 3 Department of Biomechanical Engineering, Delft University of Technology, Delft, The Netherlands; 4 Department of Rehabilitation, Medisch Spectrum Twente, Enschede, The Netherlands

**Keywords:** Spinal cord injury, Robotic gait rehabilitation, Locomotor training, Impedance control

## Abstract

**Background:**

There is increasing interest in the use of robotic gait-training devices in walking rehabilitation of incomplete spinal cord injured (iSCI) individuals. These devices provide promising opportunities to increase the intensity of training and reduce physical demands on therapists. Despite these potential benefits, robotic gait-training devices have not yet demonstrated clear advantages over conventional gait-training approaches, in terms of functional outcomes. This might be due to the reduced active participation and step-to-step variability in most robotic gait-training strategies, when compared to manually assisted therapy. Impedance-controlled devices can increase active participation and step-to-step variability. The aim of this study was to assess the effect of impedance-controlled robotic gait training on walking ability and quality in chronic iSCI individuals.

**Methods:**

A group of 10 individuals with chronic iSCI participated in an explorative clinical trial. Participants trained three times a week for eight weeks using an impedance-controlled robotic gait trainer (LOPES: LOwer extremity Powered ExoSkeleton). Primary outcomes were the 10-meter walking test (10MWT), the Walking Index for Spinal Cord Injury (WISCI II), the six-meter walking test (6MWT), the Timed Up and Go test (TUG) and the Lower Extremity Motor Scores (LEMS). Secondary outcomes were spatiotemporal and kinematics measures. All participants were tested before, during, and after training and at 8 weeks follow-up.

**Results:**

Participants experienced significant improvements in walking speed (0.06 m/s, *p* = 0.008), distance (29 m, *p* = 0.005), TUG (3.4 s, *p* = 0.012), LEMS (3.4, *p* = 0.017) and WISCI after eight weeks of training with LOPES. At the eight-week follow-up, participants retained the improvements measured at the end of the training period. Significant improvements were also found in spatiotemporal measures and hip range of motion.

**Conclusion:**

Robotic gait training using an impedance-controlled robot is feasible in gait rehabilitation of chronic iSCI individuals. It leads to improvements in walking ability, muscle strength, and quality of walking. Improvements observed at the end of the training period persisted at the eight-week follow-up. Slower walkers benefit the most from the training protocol and achieve the greatest relative improvement in speed and walking distance.

## Background

Spinal cord injury (SCI) affects 10.4 [1] to 83 [2] per million individuals per year (in developed countries), leading to an estimated prevalence that ranges between 223 and 755 per million inhabitants [3]. Learning to walk again is a major goal during SCI rehabilitation [4,5]. Generally, more than 50 percent of patients have motor incomplete lesions (iSCI) [3], of which around 75 percent regain some ambulatory function [6]. Still, many iSCI individuals experience limited hip flexion during swing phase and insufficient knee stability during the stance phase. Consequently, these individuals walk slower and often remain reliant on assistive devices. Over the last decades, many rehabilitation strategies have been explored to improve functional outcomes. Most are based on evidence suggesting that task-specific and intensive training, consisting of repetitive active movements and providing appropriate afferent feedback, engages spinal and supraspinal circuits, promoting neural plasticity (cortical reorganization) and increasing functional improvement [7-14].

Robotic gait-training devices have the potential to provide training sessions that support these key components. These devices reduce the labour-intensive demands on therapists and their discomfort, compared to manually assisted body-weight-supported treadmill training (BWSTT) [15]. They also enable objective monitoring of a patient’s performance and progress and reduce the between-trainer variability in terms of the applied supportive forces [16]. In the last decade, different robotic gait-training devices have been developed that are also used for other motor impairments, like stroke or multiple sclerosis. These robotic devices consist of a driven exoskeleton orthosis, like the Lokomat (Hocoma AG, Switzerland) or Auto/ReoAmbulator (HealthSouth/Motorika, USA) that drives the hip and knee joint, or driven footplates that facilitate a stepping motion like the Gait Trainer (Reha-Stim, Germany), G-EO (RehaTechnologies) or LokoHelp (LokoHelp Group, Germany).

Although these robotic gait-training devices have been on the market for more than a decade, research on their efficacy is still at an early and rather inconclusive state. On the one hand several studies showed improvements in walking ability between pre- and post-training in acute and chronic iSCI individuals who trained with the Lokomat [14,17-20] or Gait Trainer [19,21]. On the other hand, only very few randomized controlled trials (RCTs) [22-25] or other study designs [19,26], were performed to investigate whether these improvements are superior to those obtained using conventional approaches. Results from these studies, however, show contradictory results. Recent reviews have also concluded that robotic gait-training devices have not yet demonstrated clear advantages over conventional gait-training approaches in terms of effectiveness of training [27-29].

The limited effectiveness of the first-generation robotic gait-training devices could be attributed to some inherent limitations of these devices, which were mainly position-controlled. This type of assistance may promote “slacking”, where the user starts to rely on the robot to perform the movement and reduces his muscular activity [30,31]. In iSCI individuals, position-controlled robotic guidance, especially in individuals with some ability to walk, has been shown to actually reduce volitional activity (EMG and VO_2_) compared to therapist-assisted BWSTT [25,32]. For motor learning in general, active subject participation is considered a very important factor [33,34]. If, conversely, participants are encouraged to actively participate, they could be resisted by the position-controlled robot, causing abnormal alternations in muscle-activation patterns [35].

Another limiting factor of position-controlled robots is that they reduce movement variability to a minimum [36]. Kinematic variability, and the possibility to make movement errors is necessary to (re)learn any new task [37,38]. In this respect, traditional robotic gait-training devices only partly meet the requirement for task-specific, intensive, active, and variable training. In other words, they do not resemble the manual assistance provided by a therapist who is likely to be compliant, motivational, and intuitively adaptive to the needs of the individual and who inherently introduces a natural sense of variability.

This situation demonstrates the need to develop and improve control approaches that increase active participation and natural movement variability. This can be achieved by only providing assistance when needed, and not supporting the subject’s movements that are unimpaired. Technical implementation of this strategy often consists of controlling the interaction forces between the robot and the patient. Generally, these control strategies use a healthy control spatial path to define the desired motion, in combination with a “virtual wall”/force field that determines the amount of supportive force when the individual deviates from the template (impedance control). In some cases a “moving back wall” is introduced to assist the timing of the stepping pattern [39,40].

This kind of control strategy can overcome the main criticisms against robot-aided gait training by making the robot’s behaviour more flexible and adaptive to the user’s needs. That is, the stiffness of the “virtual wall”/force field can be adapted to the capabilities, progress, and current participation of the user. This allows individuals to benefit from robot-aided treadmill training throughout the different stages of their recovery. At the initial stages of recovery, the robot can take charge (high impedance), whereas at the concluding stages of recovery, the user must contribute more to the prescribed motion (low impedance). To reduce the chance of the user becoming reliant on the support, some robotic gait-training devices use adaptive (“impedance shaping”) algorithms that reduce the stiffness of the virtual wall when kinematic errors are small [41,42]. Flexibility between steps and the possibility of making small movement errors can be increased by lowering the impedance levels or by creating a “virtual tunnel”, “dead band,” or nonlinear force-field around the healthy control template [39,40].

Lowering impedance levels might also increase motivation during training sessions. At lower impedance levels, the user has more control over his gait pattern, and additional effort/voluntary movement is reflected in the gait pattern. This way, individuals are aware of their increased activity, a sensation that can positively contribute to their active involvement. These types of controllers that 1) provide more freedom of movement, 2) only focus on the impaired aspects of gait, 3) promote active participation and 4) allow online modification of the amount of assistance (either manually or automatically), are referred to as “assist-as-needed” (AAN), “cooperative”, “adaptive”, or “interactive” controllers [39-47].

Despite the potential of AAN strategies, the superiority of this approach for iSCI individuals has not been demonstrated. In animal studies, Cai et al. and Ziegler et al. showed that AAN control algorithms allow more variation between steps and result in larger walking recovery than position-control algorithms [48-50]. Despite numerous experimental robotic gait-training devices that have been developed [51], very few of the new compliant-control strategies have been tested on iSCI individuals in multisession training protocols. In single-session experiments, Emken et al. showed that iSCI individuals trained with more variability when they used their “impedance-shaping algorithm” [41]. Duschau-Wicke et al. evaluated their “patient-cooperative approach” in a single training session and showed that iSCI individuals trained with larger kinematic variability, and with larger muscle activity, compared to non-cooperative position-controlled training [40]. Schück et al. evaluated this approach in a multisession training protocol. They used the Lokomat to train two iSCI (and two stroke) individuals for four weeks, with four training sessions of 45 minutes per week, However, they did not find a relevant increase in gait speed for iSCI individuals [52].

Most studies on robotic gait training only assess walking ability. They report functional outcome measures and clinical scales, like walking speed (10-Meter Walking Test), distance (Six-Minute Walking Test), or walking ability (WISCI II). Only a few studies assess the effect of robotic gait training on walking quality, in terms of spatiotemporal and kinematic measures [23,25]. Assessment of walking quality can provide useful insights into whether gait training restores walking function by restoration of function (using more normal movement patterns) or by compensatory strategies.

The aim of this study was to evaluate the feasibility and effect of an eight-week, multi-session training protocol using an impedance-controlled gait trainer. The effect of training was assessed in terms of walking ability and walking quality. Individual assessments were used to determine which individuals were most likely to benefit from the training protocol. To evaluate if training effects were retained post-training, we performed follow-up testing eight weeks after completion of the training protocol.

## Methods

### Participants

Subjects with chronic, motor-incomplete SCI (iSCI) were recruited from Het Roessingh Centre for Rehabilitation in Enschede, The Netherlands. Inclusion criteria were iSCI sustained at least a half year prior to enrolment, age above 18 years, a stable medical condition, a physical condition that allows for three minutes of supported walking, the ability to bear their own body weight while standing, not currently enrolled in gait training therapy, and a stable dose of anti-spastic medicine during the study. Exclusion criteria were current orthopedic issues causing problems in walking or balance, the presence of other neurological disorders, a history of cardiac conditions that interfere with physical load, the absence of independent ambulation prior to SCI, chronic joint pain and inappropriate/ unsafe fit of the robotic trainer due to the participant’s body size (bodyweight > 100 kg) and/or joint contractures. All subjects provided written informed consent including permission for publication, prior to admittance to the study. The study protocol was approved by the local medical ethics committee, METC Twente (Enschede, The Netherlands).

### Experimental apparatus

#### Rehabilitation device

**Figure 1:**
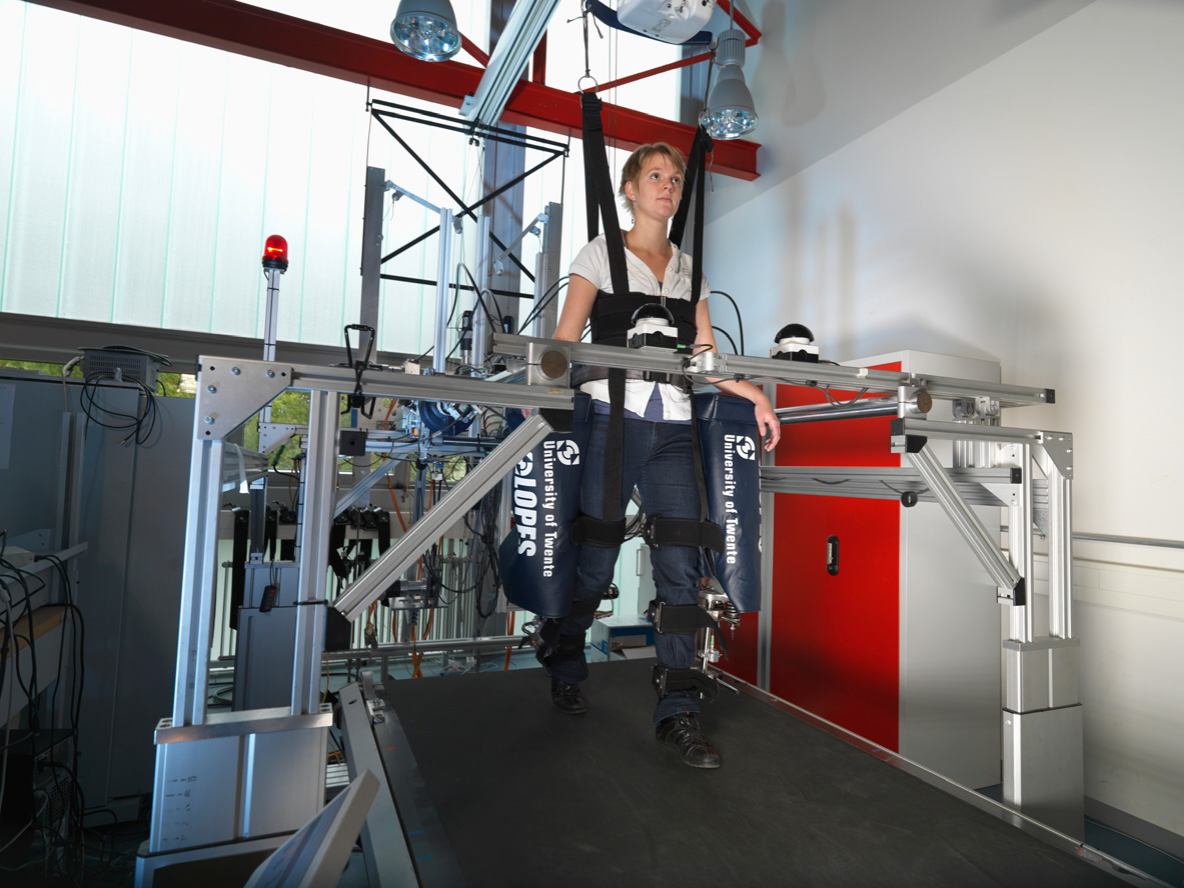
LOPES robotic gait trainer.

Gait training was done with the prototype of the LOPES gait rehabilitation robot (Figure 1). LOPES consists of a bilateral exoskeleton-type rehabilitation robot above an instrumented treadmill. It is lightweight and impedance controlled using Bowden-cable-driven series-elastic actuators. The exoskeleton offers a freely translatable (3D) pelvis, where the sideways and forward/backward motion is actuated. Furthermore, it contains two actuated rotation axes in the hip joints and one at the knee (abduction/adduction of the hip and flexion/extension of hip and knee). Passive foot lifters can be added to induce ankle dorsal flexion**,** if required. An external bodyweight-support system can relieve a definable percentage of body weight via a harness. A more detailed description of the exoskeleton design is presented in [53].

#### Joint-trajectory controller

**Figure 2:**
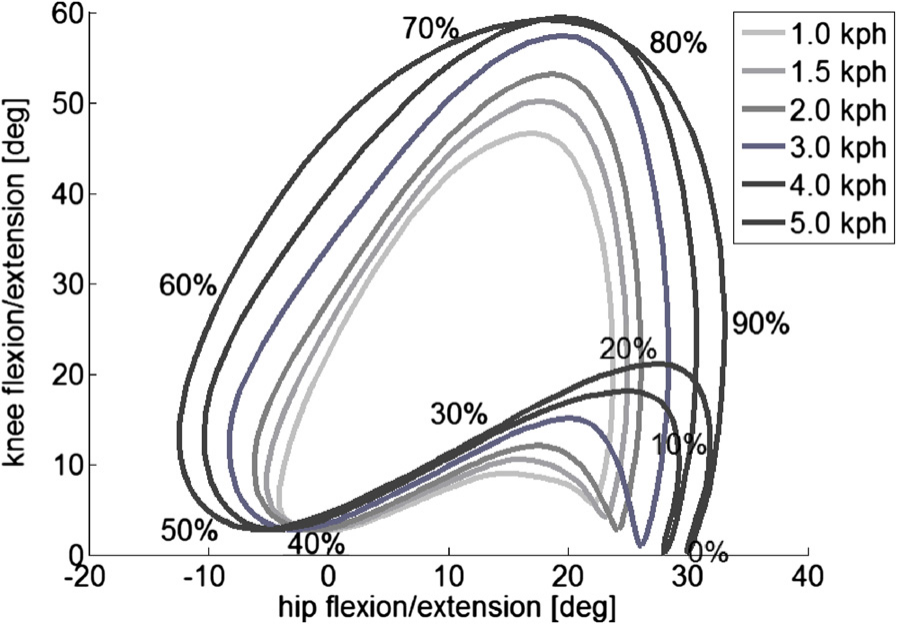
Hip and knee reference trajectories for the different walking speeds.

In this study, the amount of assistance that the participant receives is proportional to the deviation from a template or “reference walking pattern”. This reference walking pattern is derived from speed-dependent walking patterns in healthy participants. Details about the derivation of these reference patterns can be found in [54]. This method was implemented such that, when the therapist changed the treadmill speed, the joint trajectories were automatically adjusted to that specific walking speed (Figure 2).

The amount of robotic support was adjusted by changing the stiffness of the impedance controller. The impedance levels were set to a participant-specific percentage of the maximum stiffness that could be controlled by the LOPES (300 Nm/rad). In this study, the same percentage was used for hip and knee joints and for the left and right leg. To enable the participant to stay in control of his cadence, the reference walking pattern is not replayed as a function of time but is synchronized to the cadence of the participant [43].

#### Training protocol

Subjects participated in an eight-week training program. Participants trained three times per week, for a maximum of 60 minutes per session. The training period was divided in two four-week periods, with one week scheduled for clinical tests in between. During training sessions, rest intervals were introduced if required by the participant or suggested by the therapist. The first training session was used to 1) fit the LOPES to the subject, 2) let participants get used to walking in the device and 3) select their preferred walking speed.

To fit the LOPES to the subject, different anthropometric measurements were taken to adjust the exoskeleton segment lengths. Next, the subject was positioned into the LOPES and the trunk and lower extremities were secured. Three adjustable cuffs (one at the thigh, two at the shank) attached the lower extremities to the LOPES frame. Final adjustments were made to the cuffs to align the subject’s hip and knee joints with the axes of the exoskeleton joints. Bodyweight support was set at a minimal amount for each participant, preventing excessive knee flexion during stance phase or toe dragging during swing phase. Foot lifters were used in case of insufficient ankle dorsal flexion during swing phase.

**Figure 3:**
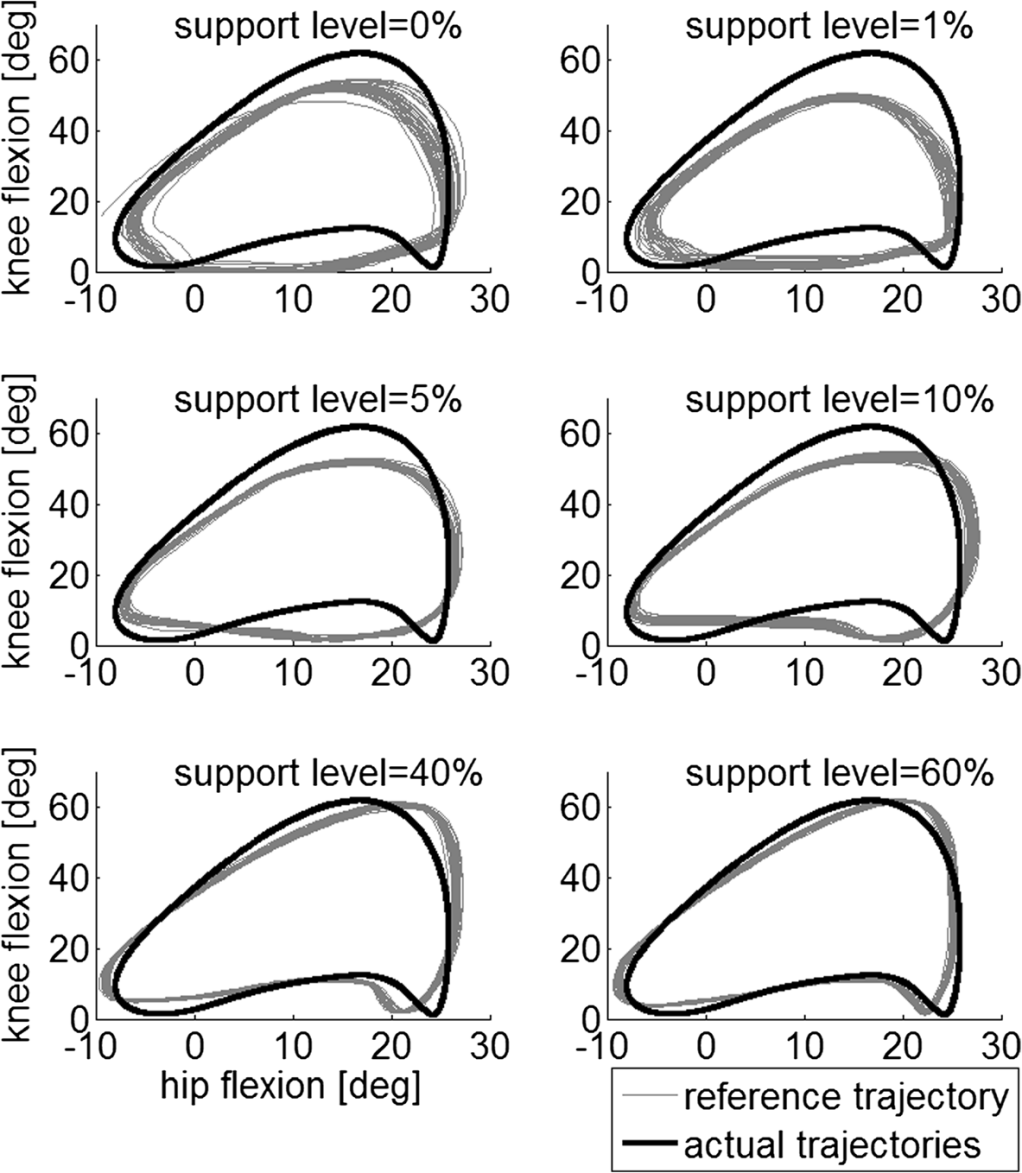
**Typical example of hip and knee reference trajectories and actual joint trajectories for a healthy subject walking at 2 km/h using different impedance levels.** Increasing the impedance levels results in a closer approximation of the reference trajectory and a reduction in the movement variability between steps. Here, the reference knee angle is enlarged by 10 percent to ensure that the robot provides support (since the healthy subject is expected to walk according to the healthy reference trajectory).

During all training sessions, the LOPES operator was paired with an experienced physical therapist. Over the training period, different parameters were adjusted to increase training intensity. Walking speed was the first parameter to be increased when possible. Subsequently, the total training time per session was increased and BWS levels were decreased. To promote active patient participation, the impedance levels of the LOPES were reduced when possible. This controller could vary between very stiff (robot-in-charge) to very flexible (patient-in-charge). Lower impedance levels also allowed more variability in the stepping trajectory (Figure 3) [41].

Adjustments of training parameters were done by the physical therapist based on the quality of walking (adequate step height during swing phase and adequate knee stability during stance phase), current physical condition (observation of breathing rate and degree of transpiration), and motivation (as verbally indicated by the participant). All changes were made in agreement with the participant. All training parameters were stored for later analysis.

### Outcome measures

#### Primary outcome measures

To assess changes in muscle strength and walking ability, clinical tests were performed before (pre), during (mid), and after (post) eight weeks of training. To examine whether the training effects were retained, we also performed a follow-up eight weeks after the completion of the training protocol.

Walking speed was measured using the 10-Meter Walk Test (10MWT). Participants were instructed to walk in a straight line at their own comfortable speed. Distance/endurance was tested with the Six-Minute Walk Test (6MWT), where participants ambulated for six minutes at their self-selected speed. The Timed Up and Go (TUG) test assessed the combination of balance during walking, gait speed, and sit-to-stand transitions. In this composite test, the patient must get up from a chair, walk 3 meters, return, and sit down again. For these three tests, participants were permitted to use braces and walking devices. The Walking Index for Spinal Cord Injury II (WISCI-II) was used to quantify the amount of assistance required during over-ground ambulation and to assess the use of assistive devices and/or orthoses. Category 0 indicates the participant could not walk or stand and category 20 indicates the participant could walk at least 10 m without assistance or use of assistive devices. All of these measures were taken according to van Hedel et al. [55]. Muscle strength was determined by the Lower Extremity Motor Scores (LEMS), utilized by the American Spinal Injury Association (ASIA). The strength of five key muscles are graded from 0 to 5 (0 indicates absence of muscle contraction and 5 is a normative active movement with full range of motion against full resistance). The cumulative score for lower extremities is between 0 and 50 [56]. All measures were recorded by an experienced physical therapist, not involved in the training.

#### Secondary outcome measures

To assess changes in gait quality, kinematic data and spatiotemporal measures were taken pre- and post-training. Gait kinematics were recorded using an optical tracking system, consisting of six infrared cameras (Vicon PlugIn Gait Model, VICON, Oxford Metrics, Oxford, UK) and reflective markers. Participants walked at their preferred speed across a 7-meter walkway approximately 10 times and were allowed to rest between rounds. Kinematic data from right and left limbs of each participant were extracted and averaged over at least 10 steps, using custom-written software (MATLAB, Mathworks Inc., Natick, MA, USA). The use of assistive walking devices and orthotic devices for safe over-ground walking was allowed (and kept constant during the pre- and post-measurements).

A total of nine parameters were extracted from the kinematic data: walking speed, cycle time, step symmetry index, step length, step width, relative stance phase duration, maximum knee flexion during the swing phase, range of motion (ROM) of the knee during the stance phase (initial- and mid-stance) and hip ROM. These parameters were used for comparison between pre- and post-training.

Cycle time was defined as the time between two consecutive heel strikes of the same leg. Range of motion of the knee during the stance phase was used to assess knee stability during the stance phase. Step width was determined as a measure of gait stability [57]. The step symmetry index was calculated according to equation 1.

(1) $$\mathrm{SI}=\frac{\mathrm{SLs}-\mathrm{SLw}}{0.5\left(\mathrm{SLs}+\mathrm{SLw}\right)}\cdot 100\%\;$$

*SLs* represents the step length of the stronger leg and *SLw* the step length of the weaker leg. Here, a symmetry index of zero indicates perfect symmetry between the two legs. Similarly to Nooijen et al. [23], the stronger leg was defined as the leg that, on average, made the largest steps during the pre-test. In all participants, the weak leg during the pre- and post-training remained the same.

The step length, relative stance phase duration, maximum knee flexion during the swing phase, ROM of the knee during the stance phase and the hip ROM were also separately calculated for the weaker and stronger leg.

#### Statistics

Measurements of walking ability were assessed pre-, mid-, and post-training and at follow-up. Because of the lack of normally distributed data (determined by Shapiro-Wilk test) and the relatively small number of participants, nonparametric statistical tests were used to detect changes throughout the training period. Statistical analysis was done on the absolute values for all measurements. To assess the effect of the training protocol on functional outcome (10MWT, 6MWT, WISCI II, TUG and LEMS), the Friedman analysis of variance by ranks was used, with *P* < 0.05. Post-hoc comparisons were performed using the Wilcoxon signed-rank test and a Bonferroni correction to account for multiple comparisons (*P* < 0.017). To assess retention of the functional level at follow-up, a Wilcoxon signed-rank test was performed to detect changes between post-training and follow-up with significance *P <* 0.05. Spearman correlation coefficients were calculated to identify possible correlations between the initial performance on the walking ability tests and the absolute change in these measures (*P <* 0.05). Measurements of walking quality (kinematic and spatiotemporal measures) were only assessed pre- and post-training. Changes in walking quality between pre- and post-training were determined with the Wilcoxon signed-rank test (P < 0.05). All statistical tests were performed with SPSS Statistics (IBM Corp., Armonk, NY, USA).

## Results

### Participants

A total of 12 participants with iSCI were included. Participant characteristics are listed in Table 1. Two participants dropped out (subjects 6 and 12). They did not complete the training due to medical reasons not related to the gait training.

**Table 1:** Descriptive information of participants

***Subject***	**Age**	**Gender**	Motor level of injury*	**ASIA class**	**Post- injury time (months)**
*1*	37	F	Th9	C	14
*2*	50	M	Th4	D	22
*3*	29	F	L2	B**	36
*4*	60	F	Th1	C	16
*5*	48	F	L2/Th12	D	122
6***	61	M	C5	D	14
*7*	56	F	L1/L2	C	14
*8*	31	M	C5	C	120
*9*	63	M	C3/C2	C	16
*10*	46	F	C5	D	41
*11*	51	M	Th12	D	62
12***	53	M	Th12	C	84
*Mean*	48,75 ±11.3				46,75 ± 41.03

*Levels separated by a “/” indicate a difference in right and left level of injury. It is noted Right/Left.

**Diagnosed with a cauda equine syndrome.

***Dropouts.

### Training parameters

**Figure 4:**
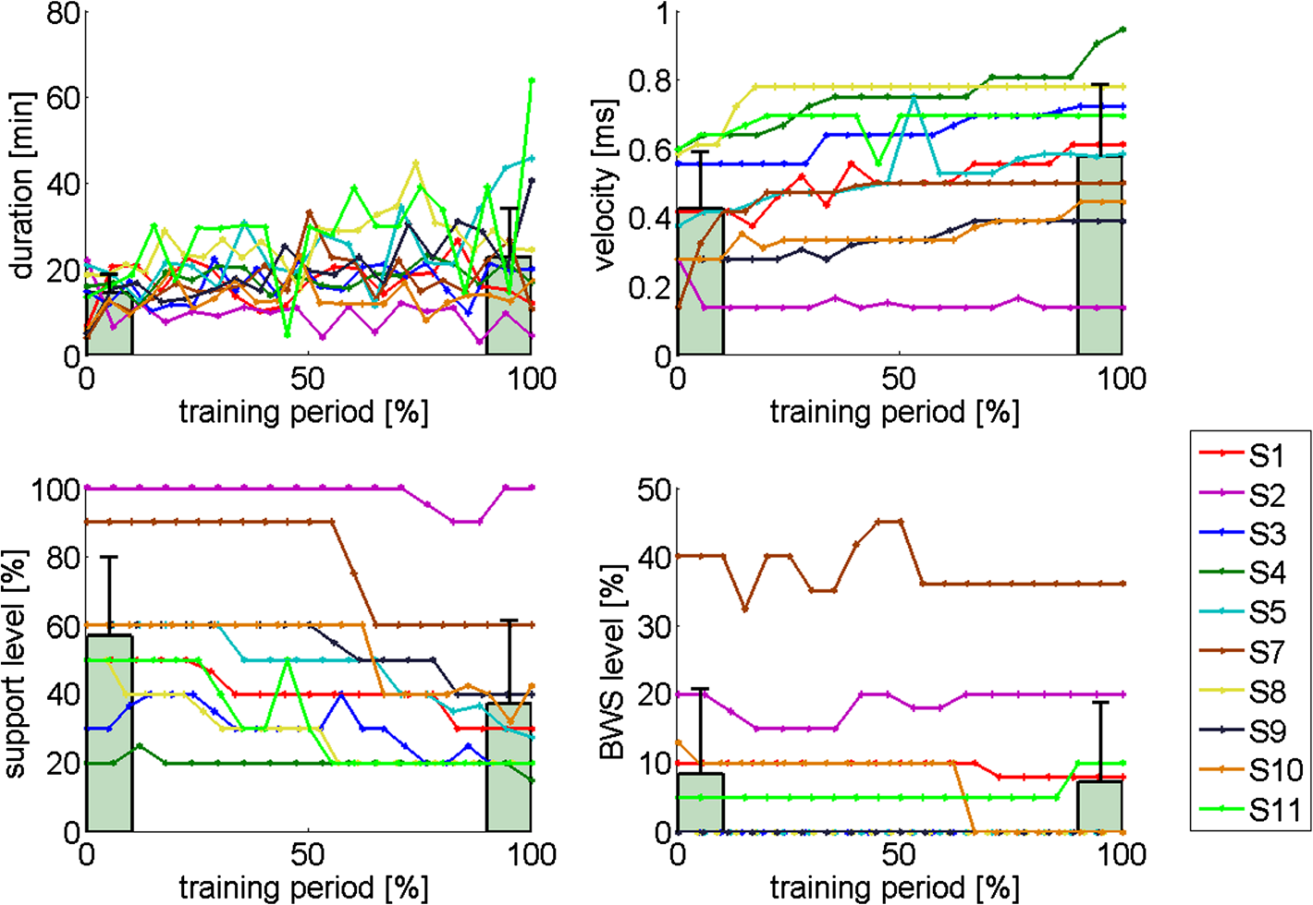
**Training parameters as a function of the training duration.** Training sessions are normalized to the total training time (0 percent start of training, 100 percent completion of training). Training duration refers to the actual total training time per session (excluding setup time and rest periods). Support levels are expressed as a percentage of the maximum stiffness that could be controlled by the LOPES (300 Nm/rad). BWS was only required in five of the 10 participants. The bars indicate the mean training parameters, averaged across participants, at the start of the training (0-10 percent) and at the end of the training period (90-100 percent). The error bars indicate the standard deviation.

Over the eight-week period, a mean number of 20.2 (range, 18- 24) training sessions were completed by the 10 participants. Due to reasons unrelated to the gait training, some participants had to cancel some training sessions. The average time ambulated during a session increased from 14.5 (± 6.1) minutes at the start of the training protocol to 22.7 (± 18.2) minutes at the end. Gait speed increased from 0.43 to 0.58 m/s. BWS was only used in five participants, and decreased from 8.5 percent to 7.4 percent. The average impedance levels/support levels decreased from 56.9 percent to 37.4 percent. Individual changes in the training parameters over the course of the training period are shown in Figure 4.

### Primary outcome measures

#### Training period

**Figure 5:**
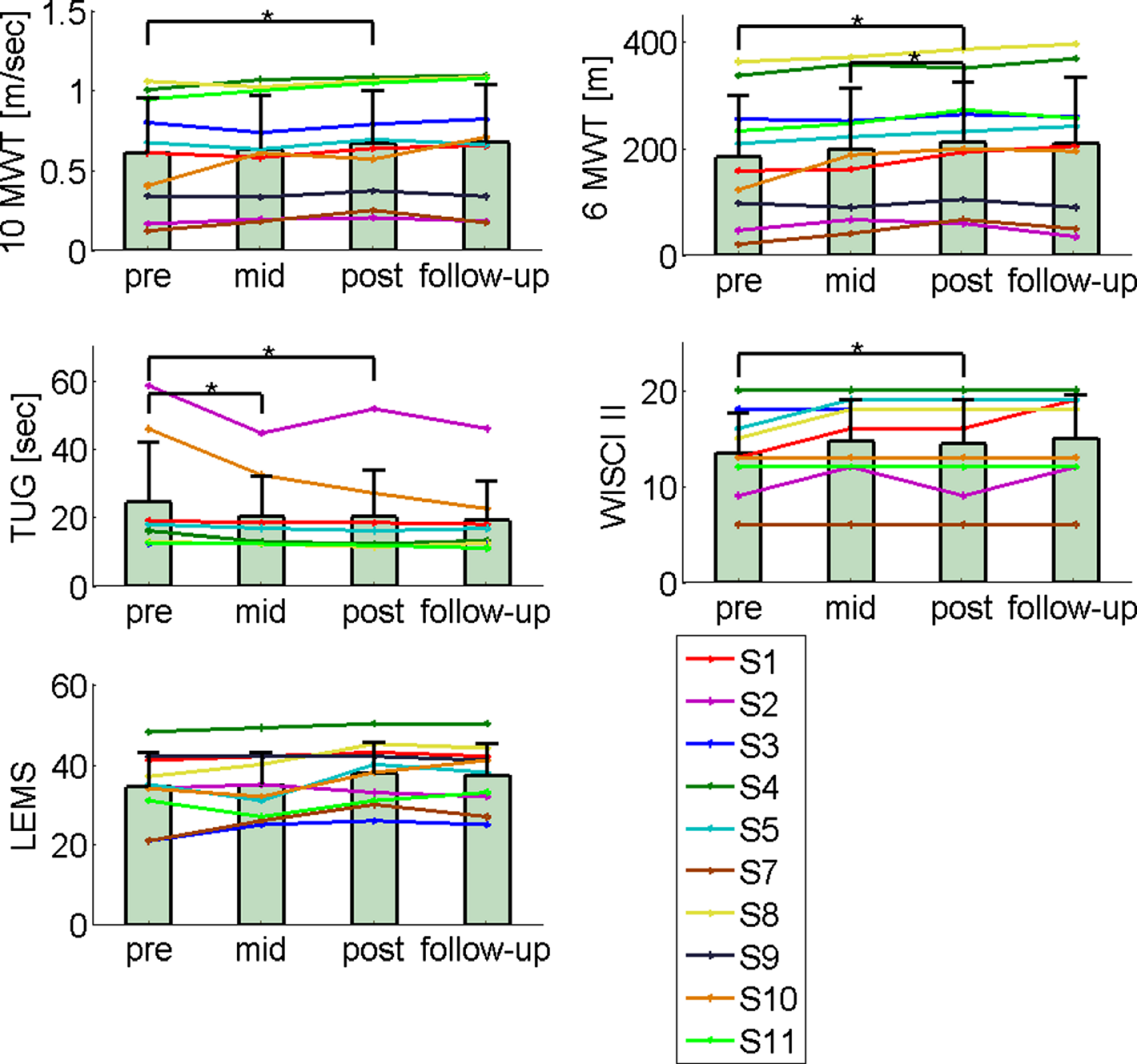
**Primary outcomes.** Measurements of walking ability were assessed pre-, mid-, and post-training and at follow-up. TUG could not be measured for subject 7 and 9. The bars indicate the mean clinical measures, averaged across participants, at each period. The error bars indicate the standard deviation.

The Friedman analysis showed a significant training effect in all walking ability and strength scores (Table 2). Subsequent, post-hoc pairwise comparisons between the different evaluation periods showed that significant improvements were primarily found between pre- and post-training. The post-hoc test between pre- and post-training revealed that eight weeks of training with LOPES resulted in significant improvements in walking speed (10MWT), distance (6MTW), TUG score, and LEMS (Table 2). No significant difference was found for the WISCI II score between pre- and post-training. Figure 5 shows the individual changes in the primary-outcome measures at the different evaluation periods.

**Table 2:** Statistical results primary outcome measures

					***Post-hoc*** **comparison**			
	**Improvement in % of subjects (pre-post)**	**Pre mean** ***(median)***	**Post mean** ***(median)***	**Main effect of time** ***p***	**Pre-mid** ***p***	**Mid-post** ***p***	**Pre-post** ***p***	**Post-follow up** ***p***
Walking speed (m/s)	90	0.61 *(0.64)*	0.67 *(0.67)*	χ^2^(2) = 8.7 0.013*	0.411	0.023	0.008*	0.797
Walking distance (m)	100	184.4 *(184)*	212.9 *(216)*	χ^2^(2) = 12.8 0.002*	0.022	0.012*	0.005*	0.507
TUG^1^ (s)	100	19.5 *(14.5)*	16.1 *(12.4)*	χ^2^(2) = 10.8 0.005*	0.017*	0.208	0.012*	0.779
WISCI-II	30	13.5 *(13)*	14.4 *(14.5)*	χ^2^(2) = 6.5 0.039*	0.046	0.317	0.083	0.157
LEMS	90	34.4 *(34.5)*	37.8 *(39)*	χ^2^(2) = 6.9 0.032*	0.210	0.258	0.017*	0.365

^*^Significant difference.

^1^The TUG was assessed in eight of 10 participants. Participants 7 and 9 were unable to stand up from the chair independently during the entire study.

#### Follow-up

All participants retained the functional level reached at completion of their training. No significant differences were found between follow-up and post-training in any of the primary outcome measures (Table 2).

#### Relationship between initial impairment levels and absolute increase

There were no significant correlations between the initial performance on walking ability tests and the absolute increase in test performance. Still, for walking speed and distance, for example, assuming an equal increase in absolute performance suggests that slower ambulators experience the greatest relative improvement. Indeed, the relative improvement in 10 MWT (ρ = -0.68, *p* = 0.04) and 6 MWT (ρ = -0.79, *p* = 0.01) showed a significant negative correlation with the initial score on these tests. The initial score on the TUG, WISI-II and LEMS did not prove to be an indicator of the relative increase in the corresponding score.

#### Secondary outcome measures

Significant changes were observed in most spatiotemporal parameters (Table 3). The maximum knee flexion during swing, the knee ROM during the stance phase, and the step width did not show significant changes. For the step length and hip ROM, the mean changes in the weak leg exceeded the changes observed in the strong leg.

**Table 3:** Statistical results secondary outcome measures

		**Increase in % of subjects (pre-post)**	**Pre mean** ***(median)***	**Post mean** ***(median)***	**Pre-post** ***p***
Walking speed (m/s)		89	0.49 *(0.57)*	0.56 *(0.64)*	0.015^*^
Cycle time (s)		11	2.24 *(1.79)*	2.04 *(1.58)*	0.032^*^
Step symmetry index (%)		22	8.46 (6.92)	4.38 (3.28)	0.021^*^
Step width (m)		33	0.11 *(0.11)*	0.10 *(0.11)*	0.114
Step length (m)	Strong and weak	89	0.44 *(0.44)*	0.47 *(0.44)*	0.017^*^
	Strong	78	0.46 *(0.46)*	0.48 *(0.47)*	0.027^*^
	Weak	100	0.42 *(0.42)*	0.46 *(0.44)*	0.007^*^
Rel. stance phase duration (%)	Strong and weak	11	74.5 *(70.6)*	72.3 *(68.8)*	0.011^*^
	Strong	11	74.6 *(71.2)*	73.0 *(69.6)*	0.028*
	Weak	0	74.4 *(70.6)*	71.5 *(68.4)*	0.008*
Maximum knee flexion (swing) (deg)	Strong and weak	56	48.6 *(49.1)*	48.4 *(48.9)*	0.859
	Strong	56	49.3 *(48.5)*	50.7 *(51.2)*	0.314
	Weak	33	47.8 *(52.2)*	46.0 *(47.9)*	0.374
Knee ROM (initial and mid stance) (deg)	Strong and weak	67	22.5 *(21.0)*	23.5 *(23.2)*	0.441
	Strong	78	23.6 *(26.8)*	26.0 *(22.8)*	0.110
	Weak	67	21.5 *(19.2)*	21.8 *(14.8)*	0.953
Hip ROM (deg)	Strong and weak	100	36.7 *(34.9)*	38,8 *(38.9)*	0.008^*^
	Strong	67	37.0 *(38.2)*	39.0 *(37.5)*	0.051
	Weak	89	36.4 *(34.5)*	38.7 *(40.3)*	0.011^*^

^*^Significant difference.

Participant 7 was excluded for analysis of kinematic and spatiotemporal data because of the use of orthotic devices, limiting accurate 3D kinematic data collection.

## Discussion

The aim of the present study was to examine the effects of an eight-week training program on the walking ability and quality in iSCI individuals, using an impedance-control strategy. In this study, we used a prototype of the LOPES gait trainer. The training protocol was tolerated well by all 10 participants and was performed without difficulties for eight weeks. Participants improved significantly on functional outcomes, muscle strength, kinematics, and spatiotemporal measures after eight weeks of LOPES training. Subsequent follow-up evaluations revealed that participants retained their training-induced functional improvements. The main improvement in kinematics occurred at the hip. The range of motion of the hip joint increased, whereas the different measures for the knee joint were unaffected by the training protocol. Participants with the most limited initial walking function showed the largest relative improvements.

### Functional outcomes

Our main findings were a significant functional improvement and an increased muscle strength. Comparing our results with those of others is hampered because of differences in robotic devices, protocols, patient characteristics, outcome measures and the number of individuals. Furthermore, most robotic gait-training devices are rapidly evolving with increasing functionalities, making robotic gait-training strategies hard to categorize.

We found significant changes in 10MWT, 6MWT, and TUG performance that were relatively small compared to other studies (Table 4). A likely explanation for this difference is the included participants. Both Alcobendas et al. [24] and Benito-Penalva et al. [19] included acute iSCI individuals. Benito et al., who included a very wide range of participants, showed that the greatest rate of improvement was seen when training started early in rehabilitation, defined as less than six months post-injury [19]. It is very likely that the improvements in these participants are partly due to underlying spontaneous recovery [58], rather than therapy effects. These findings agree with other pilot studies, showing that individuals with the smallest time since onset of injury show the largest improvements in over-ground walking ability [14,17,21]. Additionally, most studies that include sub-acute iSCI individuals also allow their participants to receive additional gait-related therapies [19,20,24], whereas these therapies for chronic individuals have stopped, effectively increasing the intensity of the training protocol.

**Table 4:** Overview of studies using robotic gait training in patients with spinal cord injury

	**Participants time since onset device**	**Training parameters (average number of sessions)**	**10 MWT Speed (m/s) (pre – post)**	**6 MWT Distance (mtr) (pre – post)**	**TUG (sec) (pre – post)**	**WISCI II (pre – post)**	**LEMS (pre – post)**
Wirz et al., 2005 [18]	N = 20 (4) Chronic Average: 70.8 months Lokomat	8 weeks; 45 min; 3-5 ×/wk; (26 sessions)	0.38 – 0.49*	121 – 165*	61 – 36*	No significant. increase	32 – 35* (N=10)
Field-Fote et al, 2011 [22]	N = 14 Chronic ≥ 12 month N = 14 Lokomat	12 weeks; 45 min; 5 ×/wk; (49 sessions)	0.17 – 0.18	50.4 – 53.7	-	-	Left leg 12.7 – 13.9 Right leg 12.9 – 14.1*
Alcobendas-Maestro et al. 2012 [24]	N = 37 (23) Sub-acute Average: 4 months Lokomat	8 weeks; 30 min; 5 ×/wk; (40 sessions)	*0.3 – 0.4*	*110 -169*	*-*	*4 – 16*	*33 – 40*
Benito-Penalva et al. 2012 [19]	N = 105 Sub-acute <6 month N = 81 6-12 month N = 8 >12 month N = 16 Lokomat (N = 39), GT (N = 66)	8 weeks; 45 min; 5 ×/wk; (40 sessions)	0.08 – 0.26*	-	-	4.0-9.2*	22.1-30.6*
Van Nunen et al. 2013 [20]	N = 18 (9) Sub-acute and chronic <12 month N = 7 >12 month N = 11 Median: 28.8 months	12 weeks; 60 min; 2 ×/wk; (24 sessions) (20-45 min)	0.09-0.15*	-	No significant. Increase (N = 6)	No significant. increase	-
Fleerkotte et al.	N = 10 Chronic Average: 45.3 months	8 weeks; 60 min; 3 ×/wk; (20 sessions) (19 min)	0.61 – 0.67*	184.4 – 212.9*	24.4 – 20.2*	13.5 – 14.4	34.4 – 37.8*

*Indicates a significant change.

N indicates the number of participants, (..) the number of individuals initially unable to walk over the full 10 m walkway.

*Median*, mean.

From the studies including chronic iSCI individuals, Nunen et al. [20] reported similar improvements in walking speed. Wirz et al. [18], who also included only chronic iSCI survivors, observed larger improvements in walking speeds. Possible explanations for their higher gains are a greater number of training sessions (26 vs. 20), longer session durations (45 vs. 19 min), and lower initial walking speeds (0.38 vs. 0.61 m/s). A lower initial walking speed possibly allows more room for improvement. Although not discussed by Nunen et al., their results indicate that participants with initial walking speeds around 0.4 m/s show the largest improvements in walking speeds. Therefore, it seems reasonable to assume that the greater effect sizes found by Wirz et al., can be explained by the initial functional level of their participants [18]. Field-Fote et al. [22], who trained chronic iSCI individuals with very low initial walking speeds, did not find any significant effects of robotic gait training on walking speed. Apparently, iSCI individuals must have a certain level of initial walking speed/function to benefit from robotic gait training.

Although the Friedman analysis showed a significant training effect on the WISCI II score, there was no significant improvement in WISCI II scores between pre-and post-training. This is in line with other studies, taking into account the types of participants. It is known that the WISCI II is more sensitive in monitoring recovery of walking capacity in iSCI subjects during the acute stage of recovery rather than the chronic stage [59]. Similar to Wirz et al. [18], Nunen et al. [20] and others [14,17,21], we did not find effect in the WISCI II score in chronic individuals, whereas Alcobendas et al. [24] and Benito-Penalva et al. [19] reported significant increases in acute patients. Improvement in LEMS scores are similar to the results found in other studies among chronic iSCI individuals [18].

### Retention

Follow-up measurements revealed that participants in our study retained the level of functional improvement measured at the end of the training period. Studies on robotic gait training in iSCI individuals rarely include a follow-up. Field–Fote et al. did perform a follow-up among 10 individuals whose improvements exceeded 0.05 m/s to assess their retention of relearned gait abilities [22]. They concluded that walking speeds declined between the conclusion of the training and follow-up, but remained above pre-training levels. However, their follow-up group included only two chronic iSCI participants who received robotic gait training, hampering a fair comparison.

It is important to note the timing of follow-up, which was, on average 20.3 months in their study and only eight weeks in ours. Although Field-Fote et al. did not find a correlation between time since the conclusion of training and the decline in walking speed, it seems likely that participants lose some of their relearned walking abilities over time, especially if they do not exploit their relearned walking abilities in daily life [60,61].

### Spatiotemporal and kinematic measures

To our knowledge, this is the first study reporting significant changes in spatiotemporal and kinematic measures associated with increased walking ability due to robotic gait training. We found significant changes in most spatiotemporal and kinematic measures after robotic gait training. Previous studies showed small increases in cadence, step and stride length, and step-length symmetry [23] or sagittal plane excursions [25], but these were not significant. In this study, most of these measures were significantly higher after training. It is important to note that Nooijen et al. [23] and Hornby et al. [25] used the LOKOMAT without the option to decrease guidance forces, as this option was unavailable on the device at the time of their study. Although both studies encouraged participants to "walk with the machine", they both state that constant guidance may minimize the voluntary effort during training and subsequently limit improvements in gait function.

In this study, improvements in spatiotemporal and kinematic measures were greater in the weaker leg. The larger improvements in step length and hip ROM in the weaker leg resulted in significant increases in symmetry between the two legs. This may indicate that gait training restores walking function by restoration of function using more normal movement patterns, rather than compensation.

Improvements in walking speed were caused by improvements in step length as well as cadence. The increased walking speed might explain some of the observed changes in other spatiotemporal measures. Here, the increase in walking speed probably explains the decrease in stance phase duration [62] and the decrease in step width [63]. Whether the increased hip flexion is enabled by an increased hip flexion strength (mean increase in LEMS score of 3,4), or is simply a consequence of the increased walking speed [64] cannot be answered.

### Intensity

Most current rehabilitation strategies focus on recovery through intense practice of a specific task. In BWSTT training, intensity depends on a combination of duration (time or number of steps), speed, training frequency, and the amount of BWS. In this study training intensity was maximized by increasing training speed and duration, and lowering the BWS levels when possible. With the development of robotic gait trainers that can potentially support the whole movement, the amount of robotic support is also an important parameter that affects training intensity. Often the precise setting of these parameters is based on a therapist’s clinical judgment and not on experimental evidence [22]. For some parameters, the effects on training outcome are known, but for many they are not. Furthermore, the interaction among the different parameters is not being investigated. For example, reducing the amount of BWS and training at higher treadmill speeds increases efferent input. This is known to affect the neural control of stepping and is suggested to promote functional recovery [11,65]. Still, the interactive effect of BWS and walking speed in individuals following SCI is unknown. Also the tradeoff between training duration and frequency remains unknown.

For robotic gait training, the optimal amount of support also remains unclear, although reducing the amount of support according to the AAN principle seems most suitable. Here, one might follow the concepts provided in the “Challenge-Point Framework” [66]. This framework states that, for each skill level, there exists an optimal level of task difficulty. When skill levels increase, further learning will be best facilitated by increasing task difficulty. In this study, task difficulty was increased by lowering impedance levels.

To gain a better understanding of the combined effect of training duration, speed, frequency, the amount of BWS and robotic support levels, these intensity parameters should be carefully reported in future studies [67]. For example, average walking speed and BWS levels in the robotic device are rarely reported in robotic gait-training studies. Additionally, often only the total session duration is reported, which does not represent the actual training time (excluding setup time and rest periods). With the increasing interest in robotic gait-training devices that have (adaptive) impedance levels, it is also advised to report impedance levels of the robot.

Among the different robotic gait-training studies, there is a great diversity in training frequency, ranging between three to five sessions per week, and duration, ranging from 30-45 minutes per session (Table 4). These parameters are often based on financial and practical reasons [20]. In this study, training frequency fell within this range but the mean training duration (19 minutes) was considerably lower. The relatively low training duration is thought to be the result of the use of the impedance control. By lowering the impedance levels when possible, the active contribution required from participants was relatively high. As a result, some participants, especially the slowest walkers, could not train for the same duration as seen in other position-controlled gait-training studies. Still, we showed that similar gains in walking ability can be accomplished with less training time. Actually, the biggest gains in walking ability were observed in slow walkers with the lowest training duration, suggesting that active participation is equally important as training duration.

### Clinical relevance

In this study, 90 percent of participants increased their walking speed on the 10MWT, 100 percent increased their distance on the 6MWT and 100 percent reduced their time on the TUG. Although this resulted in an average significant change of 0.6 m/s, 29 m and 3.4 s (Figure 5), it should be noted that there was considerable variation among subjects. Whether these improvements represent a detectable (and clinically relevant) change is debatable. The minimally detectable change (MDC), which defines the minimal amount of change required to distinguish (with 95 percent confidence) a “true” performance change from a change due to variability in performance or measurement error, is reported to be around 0.13 m/s, 45.8 m, and 10.8 s [68]. Criteria for what clinicians define as “clinically relevant” or “meaningful” can be even higher. Although MDC criteria for detecting “true” improvements are conservative, according to them, only one participant showed a “true” improvement on the 10MWT, 6MWT and TUG tests. Still, small gains in functional improvement that can lead to reduced reliance on assistive devices could be of great personal relevance to these individuals [69].

### Limitations and future perspectives

The major limitation of this study is the lack of a control group. The rationale for not including a control group was that at this stage a pilot study was set up to assess the possible effect of impedance-controlled robotic gait training, how well it can be applied, the utility of the outcome measures chosen and the variability in patient responses [67]. It was not intended to afford a basis on which to claim that this kind of training can produce greater functional improvements than those achieved through manually assisted gait training or other forms of conventional therapy. It only shows that chronic iSCI individuals still have the capacity to improve their walking function when provided with an intensive robotic gait-training program.

Patients and therapists will probably benefit the most from robotic gait-training devices during acute stages of recovery [19]. Still, in this study, all participants were chronic individuals. We included chronic individuals (>12 months) because they typically have reached a stable level of recovery [59]. The average time since onset was 46 months, suggesting that observed improvements can be attributed to the intervention rather than spontaneous recovery. That the participants reached a stable level of recovery was also confirmed by a lack of correlation between the time since onset of the injury and the relative (or absolute) increases in walking speed. Thus, to investigate the true potential of impedance-controlled gait training, acute and sub-acute individuals should also be included in future studies. However, these trials will require larger patient numbers to reach significance due to the potential for underlying spontaneous recovery [58].

Apart from time since injury [19,20,58,69,70], previous studies also showed that ASIA levels [6,19,69,71], LEMS scores [25,71,72] (for recent injuries, for chronic results vary [18,20,22]), sensation [71,73], and age [6,71] are distinguishing factors for the degree of ambulatory capacity after gait rehabilitation. Several studies purely focus on increased walking speed, which is considered to be closely related to functional ambulation [74]. Patients who start rehabilitation programs early after injury, have higher ASIA/LEMS/sensory scores, or are younger generally show greater improvements. Factors like ethology, levels of injury, or sex seem to be less predictive [19,71]. Because of the relatively small number of participants in this study, we did not perform an analysis to relate clinical improvement to patient characteristics. Future studies should carefully document these characteristics, or stratify study participants, to determine which iSCI sub-population responds better to robotic gait training. These predictors might be different for robotic gait training where age or sensation, for instance, do not seem to have a clear effect on functional outcomes [19,70].

## Conclusions

This first explorative study using an impedance-controlled robotic gait trainer shows significant improvements in functional and qualitative walking parameters after an eight-week training program in chronic iSCI individuals. We were able to provide task-specific and intensive training sessions, even for severely affected individuals, with a minimal workload on the therapist. Compared to position-controlled robotic gait-training strategies, the training duration was relatively short, whereas improvements in functional outcomes were similar. Additionally, improvements observed at the end of the training period persisted at the eight-week follow-up. The most impaired ambulators, based on their initial walking speed, benefitted most from the training protocol in relative improvements in walking speed and walking distance.

## Competing interest

The authors declare they have no competing interests.

## Authors’ contributions

BF was involved in the study design, conducting the measurements and experiments, the analysis, and the writing of the manuscript. BK carried out the experiments, collected and analyzed data, and wrote the manuscript. JB and EA contributed to the design and revision of the manuscript. HK and JR participated in the revision of the manuscript. All authors read and approved the final manuscript.
